# Molecular markers of artemisinin resistance during falciparum malaria elimination in Eastern Myanmar

**DOI:** 10.1186/s12936-024-04955-6

**Published:** 2024-05-08

**Authors:** Aung Myint Thu, Aung Pyae Phyo, Chanapat Pateekhum, Jade D. Rae, Jordi Landier, Daniel M. Parker, Gilles Delmas, Wanitda Watthanaworawit, Alistair R. D. McLean, Ann Arya, Ann Reyes, Xue Li, Olivo Miotto, Kyaw Soe, Elizabeth A. Ashley, Arjen Dondorp, Nicholas J. White, Nicholas P. Day, Tim J. C. Anderson, Mallika Imwong, Francois Nosten, Frank Smithuis

**Affiliations:** 1 Shoklo Malaria Research Unit (SMRU), Faculty of Tropical Medicine, Mahidol-Oxford Tropical Medicine Research Unit, Mahidol University Mae Sot, Bangkok, Thailand; 2 Mahidol-Oxford Tropical Medicine Research Unit (MORU), Faculty of Tropical Medicine, Mahidol University, P. O. Box 10400, Bangkok, Thailand; 3 Centre for Tropical Medicine and Global Health, Nuffield Department of Medicine, University of Oxford, Oxford, OX3 7BN UK; 4 Disease Intervention and Prevention Program, Texas Biomedical Research Institute, P. O. Box 760549, San Antonio, TX USA; 5 IRD, Aix Marseille Univ, INSERM, SESSTIM, Aix Marseille Institute of Public Health, ISSPAM, Marseille, France; 6 Department of Population Health and Disease Prevention, Department of Epidemiology & Biostatistics, University of California, Irvine, CE 92617 USA; 7 Medical Action Myanmar, Yangon, Myanmar; 8 Microbiology Laboratory, Lao-Oxford-Mahosot Hospital-Wellcome Trust Research Unit, Mahosot Hospital, Vientiane, Lao PDR; 9 Myanmar Oxford Clinical Research Unit (MOCRU), Yangon, Myanmar; 10 Department of Molecular Tropical Medicine and Genetics, Mahidol University, P. O. Box 10400, Bangkok, Thailand

**Keywords:** *P. falciparum*, Mass drug administration, Kelch13, Artemisinin resistance, Malaria elimination

## Abstract

**Background:**

Artemisinin resistance in *Plasmodium falciparum* threatens global malaria elimination efforts. To contain and then eliminate artemisinin resistance in Eastern Myanmar a network of community-based malaria posts was instituted and targeted mass drug administration (MDA) with dihydroartemisinin-piperaquine (three rounds at monthly intervals) was conducted. The prevalence of artemisinin resistance during the elimination campaign (2013–2019) was characterized.

**Methods:**

Throughout the six-year campaign *Plasmodium falciparum* positive blood samples from symptomatic patients and from cross-sectional surveys were genotyped for mutations in kelch-13—a molecular marker of artemisinin resistance.

**Result:**

The program resulted in near elimination of falciparum malaria. Of 5162 *P. falciparum* positive blood samples genotyped, 3281 (63.6%) had K13 mutations. The prevalence of K13 mutations was 73.9% in 2013 and 64.4% in 2019. Overall, there was a small but significant decline in the proportion of K13 mutants (p < 0.001). In the MDA villages there was no significant change in the K13 proportions before and after MDA. The distribution of different K13 mutations changed substantially; F446I and P441L mutations increased in both MDA and non-MDA villages, while most other K13 mutations decreased. The proportion of C580Y mutations fell from 9.2% (43/467) before MDA to 2.3% (19/813) after MDA (p < 0.001). Similar changes occurred in the 487 villages where MDA was not conducted.

**Conclusion:**

The malaria elimination program in Kayin state, eastern Myanmar, led to a substantial reduction in falciparum malaria. Despite the intense use of artemisinin-based combination therapies, both in treatment and MDA, this did not select for artemisinin resistance.

**Supplementary Information:**

The online version contains supplementary material available at 10.1186/s12936-024-04955-6.

## Introduction

Artemisinin combination therapies (ACTs) are the first-line treatment for *P. falciparum* malaria globally. In the Greater Mekong Subregion (GMS) artemisinin resistance has emerged over the past two decades. This is characterized by slow parasite clearance [1] which facilitates the selection of partner drug resistance, resulting in ACT failures [2–5]. Artemisinin resistance in *P. falciparum* threatens recent gains in malaria control and compromises malaria elimination efforts in the countries of the GMS and beyond [6]. Recently artemisinin-resistant *P. falciparum* parasites have also been reported in several African countries [7–10]. Greater efforts are needed to eliminate *P. falciparum* malaria in these foci of multidrug resistance before it becomes untreatable [11].

Myanmar has the large majority of malaria cases in the GMS, and most of the artemisinin resistant *P. falciparum* parasites. Access to health services is very difficult in the remote areas of Myanmar where malaria is most prevalent [12, 13]. To address this, in recent years several thousand community-based health workers (CHWs) have been trained and equipped with rapid diagnostic test (RDT), antimalarials, and long-lasting insecticidal nets (LLINs). Improved community access to free early diagnosis and treatment has dramatically reduced the burden of malaria in those areas where these measures have been deployed [14, 15]. However, concerns have been raised that the intense usage of artemisinin, in particular the use of these drugs in mass drug administration MDA [16–18] could fuel the emergence and spread of drug resistance in the parasite population, although the basis for this concern has been contested [19]. Here we describe the impact of the elimination campaign on the presence of artemisinin resistant *P. falciparum* parasites.

## Background

In 2013 and 2014, a large community-based malaria elimination programme was rolled out in five large rural areas (“townships”) of Kayin State in Eastern Myanmar where multi-drug resistant *P. falciparum* parasites were prevalent [3]. There were two geographically separate programmes -one led by the malaria elimination task force (METF) who were operational in Central Kayin State, and the other led by Medical Action Myanmar (MAM) who were operational in South Kayin State. A large network of CHWs was gradually introduced by both programmes to cover the remote villages in the target areas. Long-lasting insecticide treated bed-nets were distributed, and the prevalence of malaria was assessed by ultrasensitive polymerase chain reaction (uPCR) [20–22]. Selected communities with a high prevalence of asymptomatic malaria, identified by uPCR, (hereafter referred to as “hotspots”), were targeted with mass drug administration (MDA) in order to accelerate the elimination of *P. falciparum* parasites [21, 22]. As a result of these interventions, the incidence of *P. falciparum* malaria declined dramatically throughout the elimination campaign areas [21, 23].

## Methods

### Study design

We conducted a retrospective analysis of the changing prevalence of the kelch-13 molecular markers of artemisinin resistance in *P. falciparum* during the malaria elimination programmes in Kayin State, East Myanmar. This included the results from previously published work [20, 21, 24, 25].

### Study sites

The *P. falciparum* samples included in this study were collected from the community-based malaria post network, as well as cross sectional surveys malaria surveys conducted by MAM and METF. The METF program provided malaria services in the townships of Hpapun, Hlaing Bwe, Myawaddy, Kawkareik, while the MAM program provided malaria services in Kyainseikgyi township. Early diagnosis (rapid diagnostic tests) and malaria treatments were provided in the community by trained CHWs. In total there were 1477 villages in the target areas with an estimated total population of 467,535 persons i.e. 0.9% of the population of Myanmar [21, 22].

### Malaria diagnosis and treatment

Malaria posts were operated by trained CHWs who diagnosed symptomatic cases of malaria using RDT (PF/PV SD Bioline, Alere, Korea Inc.). Patients with *P. falciparum* malaria were treated with fixed dose combination tablets of 20 mg artemether and 120 mg lumefantrine (AL) (Coartem, Novartis) at an approximate dose of 1.5/9 mg/kg taken twice a day for 3 days after a meal or with oily food. They were also given a single dose of primaquine (PQ) (Remedica Ltd) of 0.25 mg base/kg in the METF program and 0.75 mg base/kg in the MAM program. In the METF program, *P. vivax* was treated with chloroquine (Remedica, Ltd) 25 mg base/kg for 3 days [22]. In the MAM program *P. vivax* was treated with 3 days chloroquine and 14 days primaquine 0.25 mg base/kg per day [21].

### Malaria surveys and mass drug administrations

Villages were surveyed to identify high *P. falciparum* malaria prevalence (“hotspots”) using uPCR for the detection of malaria parasitaemia as described previously [21, 22, 26]. In total, 315 malaria surveys were conducted by METF and MAM between 2013 and 2017. In the METF program, a village was defined as a malaria “hotspot” if the uPCR *P. falciparum* prevalence was ≥ 20%, or the combined prevalence of *P. falciparum* and *P. vivax* was ≥ 40%. In the MAM program, a village was defined as a “hotspot” if the *P. falciparum* prevalence was > 10%, or the combined prevalence of *P. falciparum* and *P. vivax* was > 30%.

The METF program treated all identified hotspots with MDA, while the MAM program conducted a cluster randomized controlled trial in which only hotspots randomized to the intervention arm received MDA. The MDA consisted of three consecutive treatment rounds administered at one-month intervals. In each round, a weight-based regimen of dihydroartemisinin 7 mg per kg and piperaquine 55 mg per kg (DP) (Eurartesim, Sigma-Tau) was given once daily for 3 days and a single low-dose of PQ was provided on the first day [22]. All consenting individuals in the village were eligible to receive MDA except for pregnant women, children under 6 months of age, and breastfeeding mothers. These interventions and results have been reported elsewhere [20, 21].

MDA intervention was conducted in 68 hotspots and 38,617 DP treatments [3] and single low dose primaquine were administered between 2014 and 2018. Asymptomatic prevalence of *P. falciparum* was substantially reduced as well as the village-level incidence of symptomatic falciparum malaria were substantially reduced following MDA [20, 21].

### Specimen collection

Samples of *P. falciparum* DNA were collected during cross-sectional malaria surveys [21, 22], and from village malaria posts and extracted from dried blood spots on filter paper (Whatman 3MM) taken from symptomatic individuals diagnosed with *P. falciparum* malaria by RDT. The collected blood spots were labeled with the village malaria post identification code (with corresponding GPS coordinates) and were then shipped to the main offices in Mae Sot, Thailand (METF), and Yangon, Myanmar (MAM) where they were stored in a dry, temperature-controlled room until molecular analysis at Mahidol University, Thailand, and the Texas Biomedical Research Institute, USA.

### Laboratory methods

We obtained *P. falciparum* kelch (K13) gene sequence data using two approaches; Sanger sequencing and Illumina sequencing. The detailed laboratory procedures are described in the supplementary materials (Text 1).

### Kelch13 mutations classification

For this analysis, *P. falciparum* genotyped results are grouped into four categories: wild type (WT), K13 mutations definitely associated with slow parasite clearance, K13 mutations definitely not associated with slow parasite clearance, and K13 mutations where the association has not been established [27].

### Data analysis

Statistical analyses were performed using Stata (Statistical Software: Release 17. StataCorp LLC) and maps were produced using GIS software (ArcGIS version 10.5). Pre- and post-MDA allele frequencies were compared using chi-square tests.

### Ethics committee reviews

The METF program was approved by the Department of Medical Research (Lower Myanmar) 73/Ethics 2014 and OxTREC (reference no. 1017-13). The other studies that provided samples were approved by the Department of Medical Research (Myanmar) Ethics/DMR/2015/113E and Ethics/DMR/2015/109E.

## Results

**Fig. 1:**
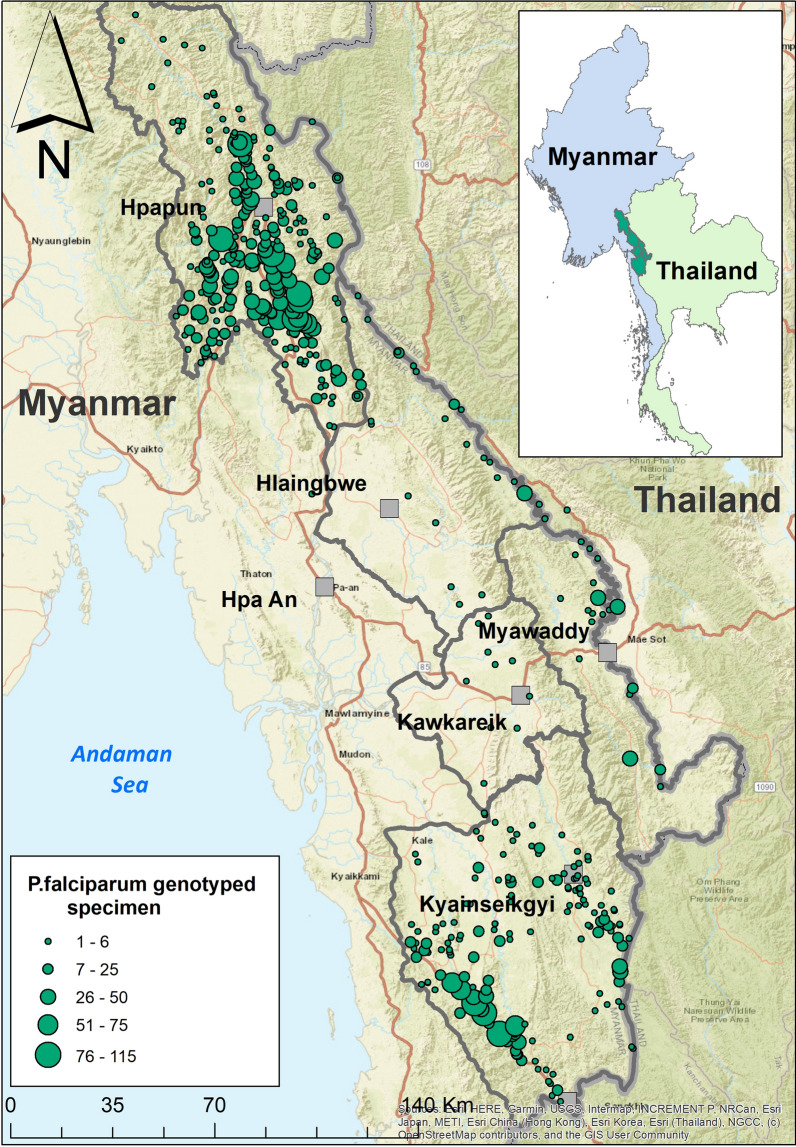
Location of collected *P.falciparum* genotyped specimen

A total of 5162 samples of *P. falciparum* DNA were collected from 543 sites between 2013 and 2019, of which 12.5% (647) were collected during malaria surveys, and 87.5% [4] were collected at the malaria posts during routine case diagnosis and treatment. The majority of isolates came from Hpapun (66.2%), followed by Kyainseikgyi (27.6%), Myawaddy (4.7%), Hlaingbwe (1.3%), and Kawkareik (0.2%) (Fig. 1).

**Fig. 2:**
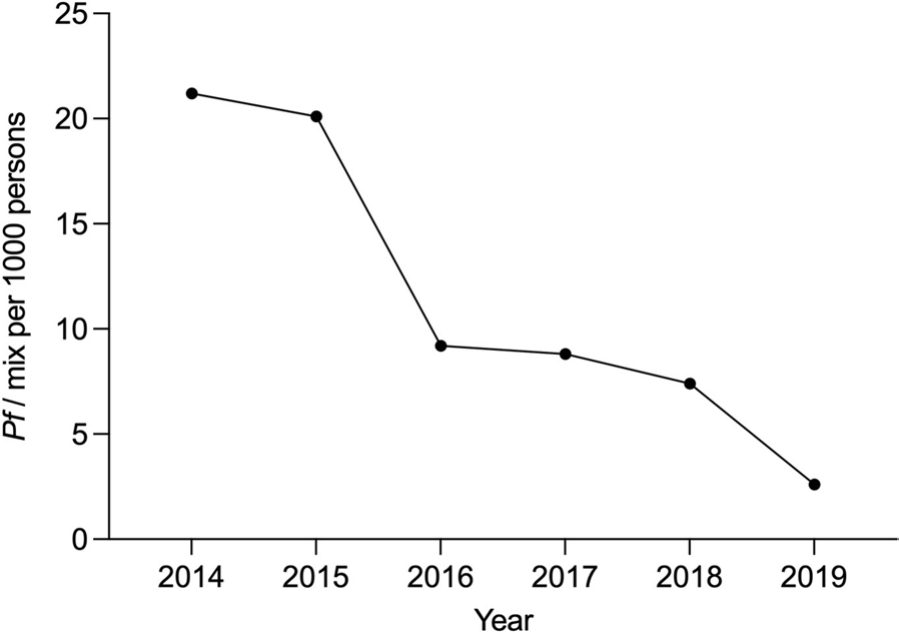
Annual symptomatic *P.falciparum/Pmix* incidence per thousand persons per year from 2014 to 2019 diagnosed and treated by malaria posts

**Fig. 3:**
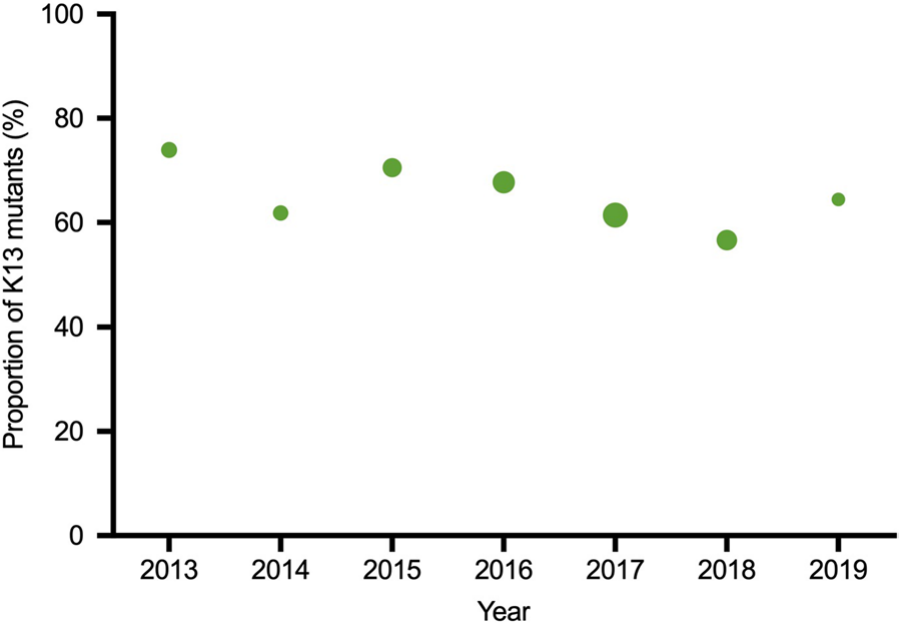
Annual proportion of *P. falciparum* isolates with K13 mutations during the six-year programmes. Circled areas are proportional to the square root of the sample size

**Fig. 4:**
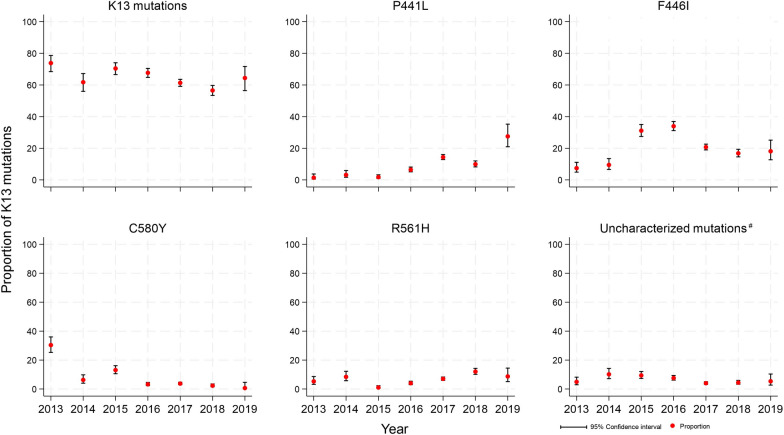
Proportion of K13 mutations from 2013 to 2019. The y-axis represents K13 mutants proportion with 95% confidence intervals while the x-axis denotes the years from 2013 to 2019. ^#^K13 mutations where the association with parasite clearance has not yet been established. The uncharacterized mutations are A621V, A626S, C469F, C469Y, C542Y, D109Y, D452E, D584V, E208K, E321K, F614L, G533A, G533D, G533S, G718S, I205T, K189T, K438N, K479I, K586E, M562I, N264I, N490H, N525Y, R265P, R528S, R529G, R575K, S423N, T192I, T535M, V193E, V494I, V534G, W611C

Falciparum malaria incidence fell dramatically (a 15-fold decline) during the intervention period from 21.2 per 1000 persons-year in 2014 to 2.6 infections per 1000 persons-year in 2019 (Fig. 2). The overall proportion of K13 mutants in *P. falciparum* isolates was 63.6%. The highest proportion was observed in 2013 (73.9%) and the lowest was in 2018 (56.6%). Overall there was a small but significant decline in the proportion of K13 mutants among the *P. falciparum* isolates (Chi-square for trend; p < 0.0001) (Fig. 3). F446I was the most common mutation, present in 22.4% (1156/5162) of all genotyped isolates. The prevalence of F446I increased from 7.4% (21/283) [95% CI 4.9–11.1] in 2013 to a peak of 33.9% (353/1040) [95% CI 31.1–36.9] in 2016, and then decreased to 18.1% (27/149) [95% CI 12.7–25.1] in 2019. P441L was the second most common mutation accounting for 9.6% (496/5,162) of all genotypes. Its prevalence increased substantially from 1.4% (4/283) [95% CI 0.5–3.7] in 2013 to 27.5% (41/149) [95% CI 20.9–35.2] in 2019. In contrast, the prevalence of the previously dominant C580Y mutation [25] was 30.4% (86/283) [95% CI, 25.3–36.0] in 2013 but then fell steadily to 0.7% (1/149) [95% CI 0.1–4.6] by 2019 (Table 1, Fig. 4).

**Table 1:** Frequencies of K13 mutations from 2013 to 2019

	2013	2014	2015	2016	2017	2018	2019	Total
	N (%)	N (%)	N (%)	N (%)	N (%)	N (%)	N (%)	N (%)
Wild type	74 (26.1)	109 (38.2)	169 (29.5)	336 (32.3)	729 (38.6)	411 (43.4)	53 (35.6)	1,881 (36.4)
K13 mutations	209 (73.9)	176 (61.8)	403 (70.5)	704 (67.7)	1158 (61.4)	535 (56.6)	96 (64.4)	3,281 (63.6)
E252Q^µ^	10 (3.5)	29 (10.2)	3 (0.5)	2 (0.2)	1 (0.1)	13 (1.4)	–	58 (1.1)
P441L^µ^	4 (1.4)	9 (3.2)	10 (1.7)	67 (6.4)	271 (14.4)	94 (9.9)	41 (27.5)	496 (9.6)
F446I^µ^	21 (7.4)	27 (9.5)	178 (31.1)	353 (33.9)	391 (20.7)	159 (16.8)	27 (18.1)	1156 (22.4)
G449A^µ^	–	9 (3.2)	28 (4.9)	76 (7.3)	139 (7.4)	84 (8.9)	6 (4.0)	342 (6.6)
N458Y^µ^	6 (2.1)	–	–	–	1 (0.1)	–	–	7 (0.1)
M476I^µ^	17 (6.0)	16 (5.6)	9 (1.6)	26 (2.5)	42 (2.2)	5 (0.5)	–	115 (2.2)
A481V^µ^	1 (0.4)	–	–	–	–	–	–	1 (0.0)
N537I^µ^	3 (1.1)	–	19 (3.3)	11 (1.1)	13 (0.7)	–	–	46 (0.9)
G538V^µ^	20 (7.1)	4 (1.4)	3 (0.5)	3 (0.3)	5 (0.3)	–	–	35 (0.7)
R539T^µ^	–	1 (0.4)	1 (0.2)	–	–	–	–	2 (0.0)
P553L^µ^	–	–	1 (0.2)	8 (0.8)	5 (0.3)	–	–	14 (0.3)
R561H^µ^	15 (5.3)	24 (8.4)	6 (1.0)	42 (4.0)	133 (7.0)	114 (12.1)	13 (8.7)	347 (6.7)
P574L^µ^	9 (3.2)	8 (2.8)	15 (2.6)	3 (0.3)	8 (0.4)	2 (0.2)	-	45 (0.9)
C580Y^µ^	86 (30.4)	18 (6.3)	75 (13.1)	34 (3.3)	71 (3.8)	22 (2.3)	1 (0.7)	307 (6.0)
P667T^µ^	–	–	–	–	2 (0.1)	–	–	2 (0.0)
A675V^µ^	3 (1.1)	2 (0.7)	1 (0.2)	–	–	–	–	6 (0.1)
Uncharacterized mutations^#^	14 (4.9)	29 (10.2)	54 (9.4)	79 (7.6)	76 (4.0)	42 (4.4)	8 (5.4)	302 (5.9)

^µ^Slow parasites clearance K13 mutants

^#^K13 mutations of which the association with parasite clearance has not yet been established. The uncharacterized mutations are A621V, A626S, C469F, C469Y, C542Y, D109Y, D452E, D584V, E208K, E321K, F614L, G533A, G533D, G533S, G718S, I205T, K189T, K438N, K479I, K586E, M562I, N264I, N490H, N525Y, R265P, R528S, R529G, R575K, S423N, T192I, T535M, V193E, V494I, V534G, W611C

### Kelch13 mutations distribution by area

#### Hpapun township

**Fig. 5:**
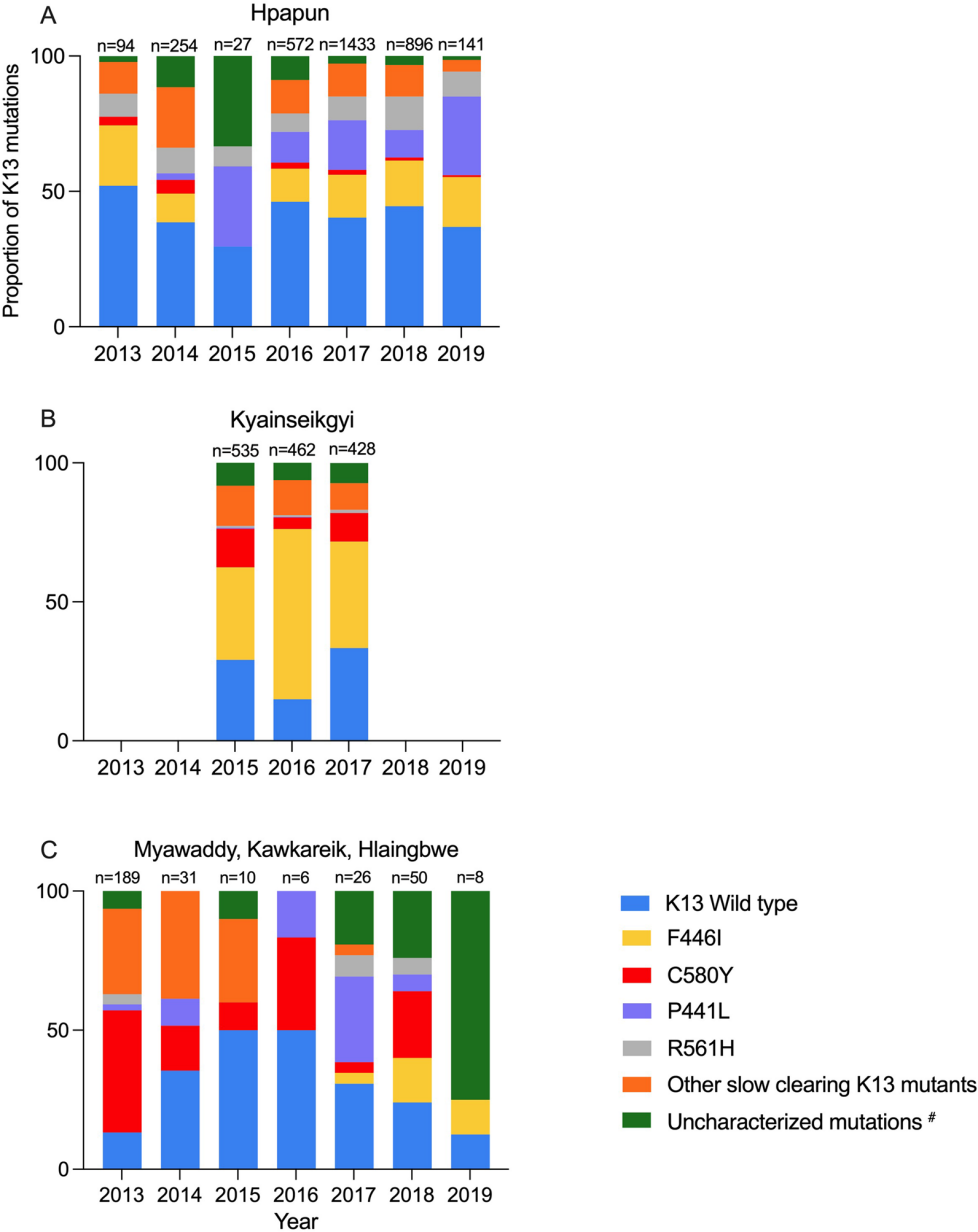
Proportion of K13 mutations from 2013 to 2019. **A** Hpapun, **B** Kyainseikgyi and **C** Myawaddy, Kawkareik and Hlaingbwe. ^#^K13 mutations where the association with parasite clearance has not yet been established. The uncharacterized mutations are A621V, A626S, C469F, C469Y, C542Y, D109Y, D452E, D584V, E208K, E321K, F614L, G533A, G533D, G533S, G718S, I205T, K189T, K438N, K479I, K586E, M562I, N264I, N490H, N525Y, R265P, R528S, R529G, R575K, S423N, T192I, T535M, V193E, V494I, V534G, W611C

K13 mutations were present in 57.6% (1969/3417) of the isolates collected in Hpapun from 2013 to 2019. The prevalence of K13 mutations was lowest (47.9%; 45/94) in 2013 and the highest (70.4%; 19/27) in 2015 (Fig. 5A). A total of 31 different K13 mutations were identified, of which 4 mutations (F446I, P441L, R561H, G449A) accounted for 46.3% (1,581/3,417) of all genotypes (Additional file 3: Table S1A). The F446I mutation was most common (15.3%) and declined in proportion slightly from 22.3% (21/94) [95% CI 15.0–31.9] in 2013 to 18.4% (26/141) [95% CI 12.9–25.7] in 2019. The P441L mutation was not detected in 2013 and was infrequent in 2014 (2.4%, 6/254) [95%CI 1.1–5.2] but then increased to become the most common K13 mutation (29.1%; 91/896) [95% CI 22.2–37.1] by 2019. C580Y was detected at low frequencies which declined from 3.2% (3/94) [95% CI 1.0–9.4] in 2013 to 0.7% (1/141) [95% CI 0.1–4.9] in 2019. (Additional file 1: Fig. S1A).

#### Kyainseikgyi township

In Kyainseikgyi K13 mutants accounted for the majority of the genotyped isolates (74.2%; 1057/1,425) collected from 2015 to 2017, during the implementation of an MDA cluster randomized trial. The prevalence of K13 mutations decreased slightly from 70.8% (379/535) [95% CI 66.8–74.5] in 2015 to 66.6% (285/428) [95% CI 42.0–70.9] in 2017 (Additional file 1: Fig. S1B). Overall F466I was the dominant mutation, accounting for 43.9% (625/1425) of the K13 mutant genotypes. The C580Y mutation was the 2nd most common detected mutation (9.6%; 137/1425) (Additional file 3: Table S1B).

#### Myawaddy, Hlaingbwe and Kawkareik

In these 3 townships, K13 mutations were present in 79.7% (255/320) of isolates. The proportion of C580Y mutants was highest in 2013, accounting for 43.9% (83/189) [95% CI 37.0–51.1] of isolates but it then declined to 24.0% (12/50) [95% CI 14.1–37.7] by 2018 (Fig. 5C). By 2019 malaria had declined substantially. Only 7 K13 mutations were identified of which 6 were uncharacterized mutations (Additional file 3: Table S1C).

### Impact of mass drug administration on the proportion of K13 mutations

A total of 1280 genotypes were collected from 69 MDA villages. Of these, 36.5% (467/1280) were collected before MDA and 63.5% (813/1280) after MDA was administered (Table 2, Additional file 2: Fig. S2). Collection of samples before MDA was done over a shorter period (median 7 months; IQR 2–12 months) than after MDA (median 14 months; IQR 7–21 months).

**Table 2:** Frequencies of K13 mutations obtained before and after MDA

	Pre-MDA	Post-MDA	Total	P value**
	N (%)	N (%)	N (%)	
Wild type	185 (39.6)	355 (43.7)	540 (42.2)	Ref
K13 mutations	282 (60.4)	458 (56.4)	740 (57.8)	0.158
E252Q^µ^	13 (2.8)	14 (1.7)	27 (2.1)	0.14
P441L^µ^	28 (6.0)	87 (10.7)	115 (9.0)	0.039
F446I^µ^	50 (10.7)	187 (23.0)	237 (18.5)	< 0.001
G449A^µ^	22 (4.7)	48 (5.9)	70 (5.5)	0.638
N458Y^µ^	–	2 (0.3)	2 (0.2)	–
M476I^µ^	9 (1.9)	12 (1.5)	21 (1.6)	0.42
N537I^µ^	1 (0.2)	–	1 (0.1)	–
G538V^µ^	2 (0.4)	20 (2.5)	22 (1.7)	0.014
P553L^µ^	3 (0.6)	2 (0.3)	5 (0.4)	0.228
R561H^µ^	42 (9.0)	26 (3.2)	68 (5.3)	< 0.001
P574L^µ^	7 (1.5)	5 (0.6)	12 (0.9)	0.083
C580Y^µ^	43 (9.2)	19 (2.3)	62 (4.8)	< 0.001
P667T^µ^	–	2 (0.3)	2 (0.2)	–
A675V^µ^	2 (0.4)	1 (0.1)	3 (0.2)	0.24
Uncharacterized mutations^#^	60 (12.9)	33 (4.1)	93 (7.3)	< 0.001

Specimen collection period, Pre-MDA (median 7 months; IQR 2–12 months) and post-MDA (median 14 months; IQR 7–21 months)

^µ^slow parasites clearance K13 mutants

^#^K13 mutations of which the association with parasite clearance has not yet been established

^**^P-value from Chi-squared test for the K13 mutations before and after MDA

**Fig. 6:**
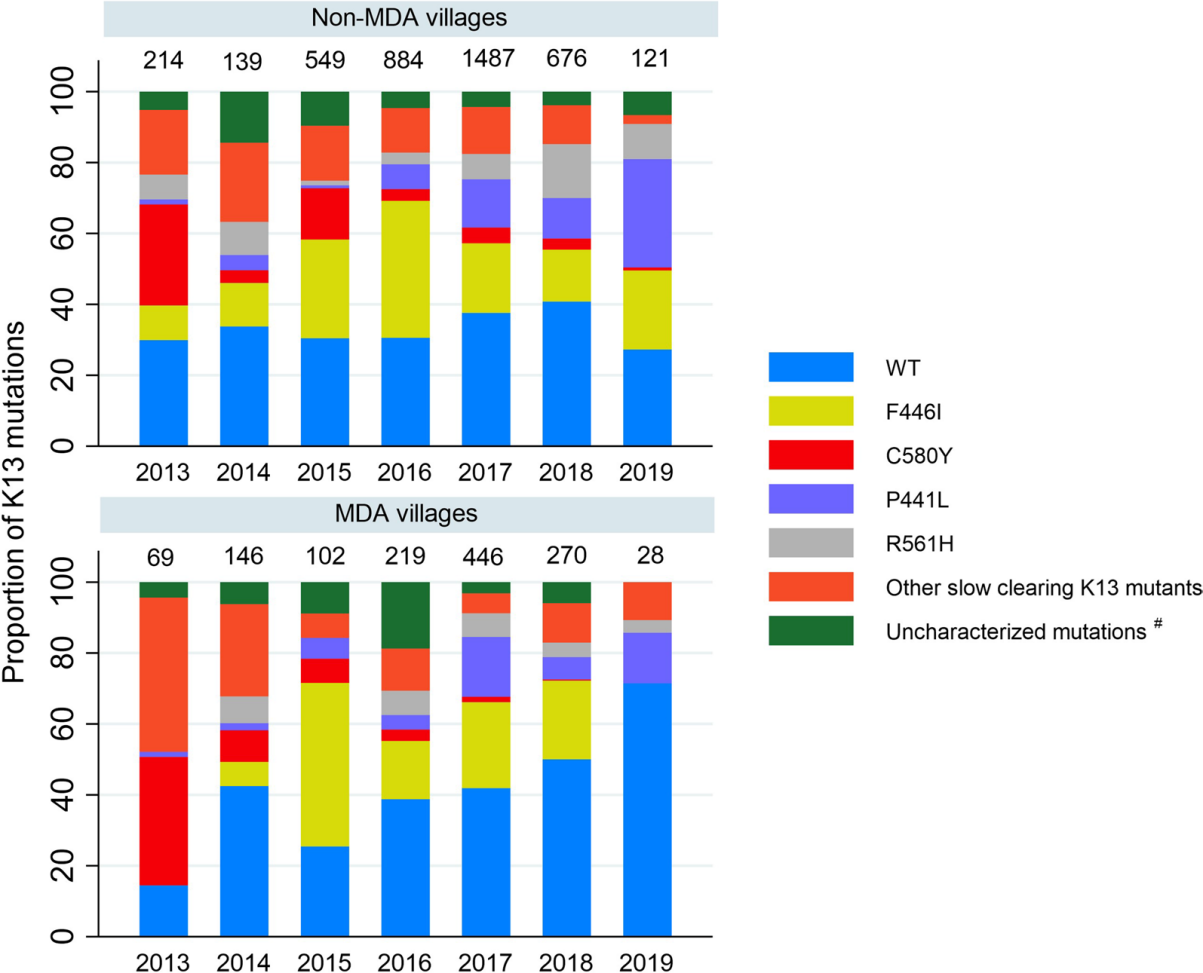
Proportion of K13 mutations comparison in MDA and non-MDA villages from 2013 to 2019. ^#^Uncharacterized mutations are K13 mutations where the association with parasite clearance has not yet been established

Comparing samples gathered before and after MDA the proportion of F446I mutations increased from 10.7% (50/467) to 23.0% (187/813), (p < 0.001), the proportion of P441L mutations increased from 6.0% (28/467) to 10.7% (87/813) (p = 0.039) and the proportion of C580Y mutants decreased from 9.2% (43/467) to 2.3% (19/813) (p < 0.001), (Table 2). However, overall the proportion of isolates with K13 mutations was similar before MDA (60.3%; 282/467) and after MDA (56.3%; 458/813) (p = 0.158). In 487 villages where MDA was not delivered (which by design had less malaria than the MDA villages) the overall proportion of *P. falciparum* isolates with K13 mutants did not change significantly between 2013 (70.1%, 150/214) and 2019 (73%, 88/121) (p = 0.610). As in the MDA villages, the proportion of F446I mutants increased from 9.8%, (21/214) in 2013 to 22.3%, (27/121) in 2019 (p < 0.001), the proportion of P441L mutations increased from 1.4% (3/67) in 2013 to 30.6% (37/121) in 2019 (p < 0.001) and the proportion of C580Y mutations decreased from 28.5% (61/214) in 2013 to 0.8% (1/121) in 2019 (p < 0.001). (Fig. 6). There was no difference in the prevalence of K13 mutations in villages where MDA was deployed to those in contemporaneous areas where MDA was not deployed.

## Discussion

Artemisinin resistance in *Plasmodium falciparum* was first identified in western Cambodia nearly 20 years ago, and it is now widespread in the Greater Mekong subregion [1, 25]. Mutations mainly in the propeller region of the K13 gene cause artemisinin resistance [28], and this manifests as slow parasite clearance following treatment [2]. Other genetic factors also contribute to the phenotype of reduced ring stage antiparasitic activity and thus slow parasite clearance following treatment with artemisinin drugs [29]. Resistance to the artemisinin component of ACTs leaves the partner drugs with a higher residual load and thus a greater probability of selecting partner drug resistance [30, 31]. This has been documented both for mefloquine and piperaquine containing ACTs [3, 5, 32]. Among several independent emergences with different K13 mutations a single dominant lineage with slow parasite clearance (K13 C580Y) was selected and spread widely over Cambodia and surrounding areas in Vietnam, Laos and East Thailand [33]. This dominant parasite lineage was linked with high dihydroartemisinin-piperaquine treatment failure rates [4].

Meanwhile in Myanmar, which has the vast majority of malaria cases in the Greater Mekong subregion (GMS), different lineages emerged and then spread over wide distances [33]. This compromised the artesunate-mefloquine combination [3] but so far has not compromised the national first-line recommended antimalarial treatment artemether-lumefantrine (although there are few recent data) [34]. When artemisinin resistance was recognized first it seemed that the best containment strategy was to try and eliminate all falciparum malaria from the affected regions. To complete this rapidly mass drug administrations would be needed in focal areas with malaria hotspots, and this would require use of dihydroartemisinin-piperaquine and single low-dose primaquine. This was regarded as a risk for the further selection and spread of artemisinin resistance. The falciparum malaria elimination campaign in Eastern Myanmar described in this study resulted in a rapid decline in *P. falciparum* malaria incidence (Fig. 2) [20, 21, 23]. Critically, while there were changes in the proportions of different K13 genotypes, there was no overall variation in the prevalence of K13 mutations despite the widespread deployment of artemisinin-based combination therapy both in treatment and MDA campaigns.

The parasite lineage with the K13 C580Y allele dominated initially on the Thailand-Myanmar border in 2013 [3, 34, 35], but this declined over the six years study period and has now almost disappeared in this region. Interestingly a C580Y mutant lineage of different origin also dominated in the Eastern GMS and also has declined in prevalence relative to other K13 genotypes in recent years [25]. Meanwhile, in Eastern Myanmar the proportions of parasites with F446I and P441L mutations increased. In addition, the G449A and R561H mutations also became established in the area, albeit to a lesser extent. Other K13 mutations were identified at low frequencies. The F446I mutation confers less slowing of parasite clearance [2], but the reasons why some mutations increase in relative prevalence is not well characterized—and may relate to other genetic factors increasing relative fitness which are unrelated to artemisinin resistance. Among uncharacterized mutations in Kelch13 gene, 68% of these mutants were reported in Africa, Asia, Americas and Oceania according to the WWARN artemisinin Molecular Surveyor (http://www.wwarn.org/molecular/surveyor/k13).

In the villages where MDA was conducted, we found no evidence that MDA had selected for artemisinin resistance in the few remaining parasites. It is commonly stated that MDA selects for drug-resistant parasites, but this depends on how effective the MDA is. Poorly implemented MDA with low coverage or inadequate regimens might fail to eliminate the parasites. This failure might create a selective pressure where resistant strains survive and multiply, potentially leading to an increased of artemisinin resistance in the long run.

In this elimination campaign MDA interventions resulted in a significant reduction in *P. falciparum* incidence through high MDA coverage with an effective treatment regimen among targeted hotspots [20, 21]. MDA generally encounters relatively low parasite densities in infections that have already been controlled by host immunity, and this increases the probability of successful parasite clearance. During MDA, all parasite carriers are treated, and the number of *P. falciparum* infections with high parasite biomass is reduced, resulting in fewer courses of ACT treatment needed to treat symptomatic higher burden infections. This reduces the risk of resistance selection [36]. The deployment of different ACT regimens for routine treatment and for MDA is an additional factor which reduces the chance of selecting resistant parasites [37]. The high level of participation of the villagers in the elimination programme, facilitated by effective community engagement [38], and the absence of other sources of antimalarials, likely played an additional role in preventing the increase of artemisinin resistance in this area.

This study has several limitations. It is a largely observational experience. There were no K13 data on the period before the establishment of the malaria post/ community health worker network in Eastern Myanmar. Other molecular markers of antimalarial drug resistance were not assessed, although molecular markers of lumefantrine resistance (other than Pfmdr1 amplification) are not well established [25, 39]. MDA interventions quickly followed establishment of the malaria posts in the “hotspot” villages, and so there was shorter follow-up time and there were fewer *P. falciparum* specimens collected in pre-MDA period than in the post-MDA period. This may have resulted in an underestimate of the presence of some mutant alleles before MDA. Most of the specimens were collected from febrile patients who presented to the malaria posts with a positive malaria HRP2 antigen based rapid diagnostic test. Theoretically, this means that infections caused by parasites that carry histidine rich proteins deletion mutation (HRP2) could have been missed. However, the rate of *Pf*HRP2 deletion has been reported to be very low in Myanmar [40, 41].

Continuing surveillance of genetic markers for both artemisinin and partners drugs resistance is warranted in this area.

## Conclusion

This elimination campaign, was successful in reducing the incidence of *P. falciparum*. Despite the intense use of artemisinin-based combination therapies, both in treatment and MDA, it did not select for further artemisinin resistance.

### Supplementary Information

**Additional file 1: Figure S1.** Proportion of K13 mutations by townships from 2013 and 2019. (A) Hpapun (B) Kyainseikgyi (C) Myawaddy, Hlaingbwe and Kawkareik. The y-axis indicates K13 mutant proportion with 95% confidence intervals.

**Additional file 2: Figure S2.** Comparison of the proportion K13 mutations before and after MDA. # Uncharacterized mutations are K13 mutations where the association with parasite clearance has not yet been established.

**Additional file 3: Table S1A.** K13 mutations frequency in Hpapun township from 2013 to 2019. **Table S1B.** K13 mutations frequency in Kyainseikgyi township from 2015 to 2017. **Table S1C.** K13 mutations frequency in Myawaddy, Kawkareik and Hlaingbwe townships from 2013 to 2019. **Table S2.** Status of uncharacterized K13 mutants in other contexts.

## Data Availability

All relevant data are within the manuscript. Upon reasonable request via the MORU website or from MORU data sharing committee datasharing@tropmedres.ac the raw data set will be available.
