# Supplementary figures and images for: Molecular markers of artemisinin resistance during falciparum malaria elimination in Eastern Myanmar
Source: Malar J. 2024 May 8;23:138. doi: 10.1186/s12936-024-04955-6 (PMC11078751; doi:10.1186/s12936-024-04955-6)

Proportion of K13 mutations

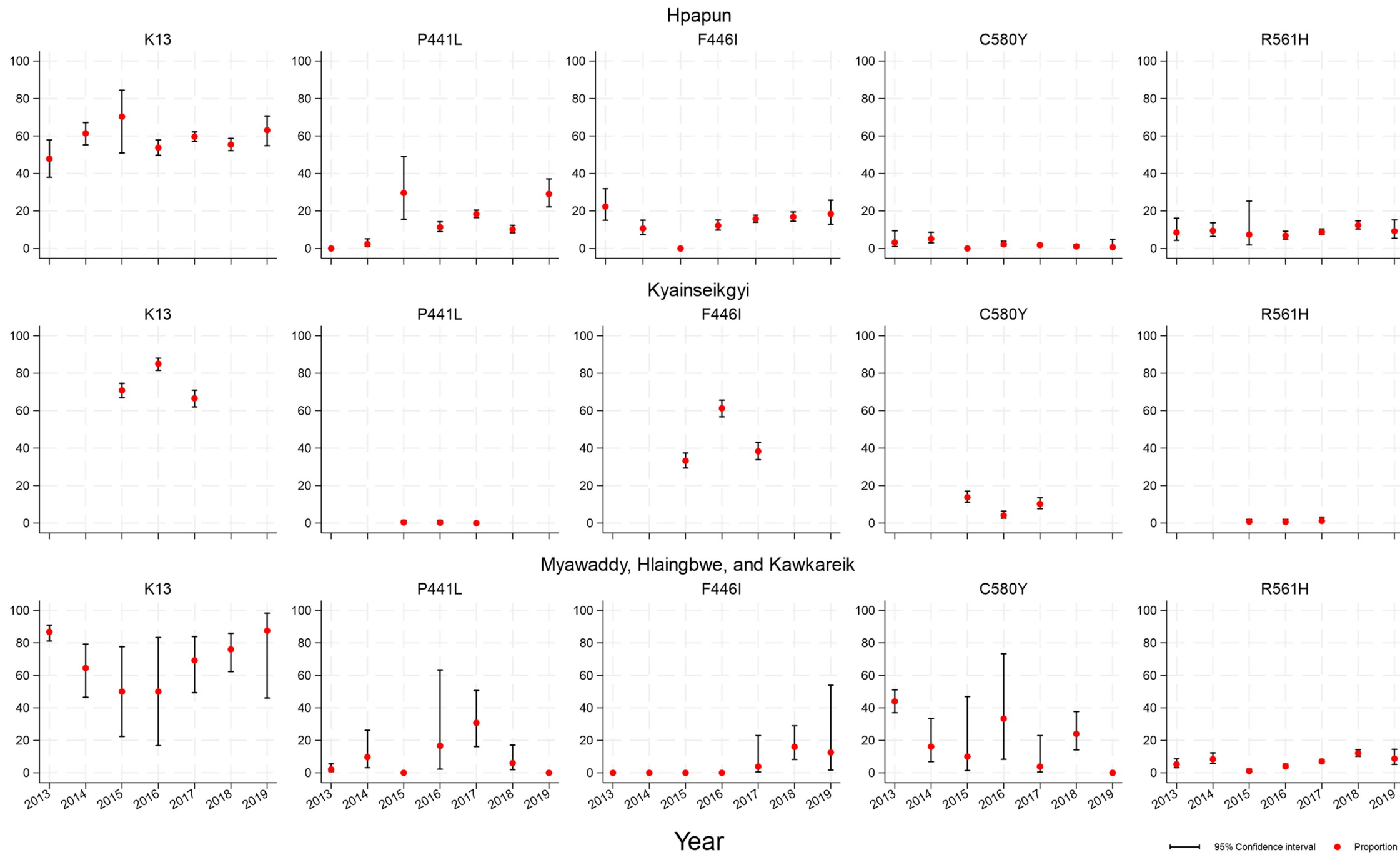

Supplement: Supplementary file 1 — Additional file 1: Figure S1. Proportion of K13 mutations by townships from 2013 and 2019. (A) Hpapun (B) Kyainseikgyi (C) Myawaddy, Hlaingbwe and Kawkareik. The y-axis indicates K13 mutant proportion with 95% confidence intervals. [file 12936_2024_4955_MOESM1_ESM.pdf]

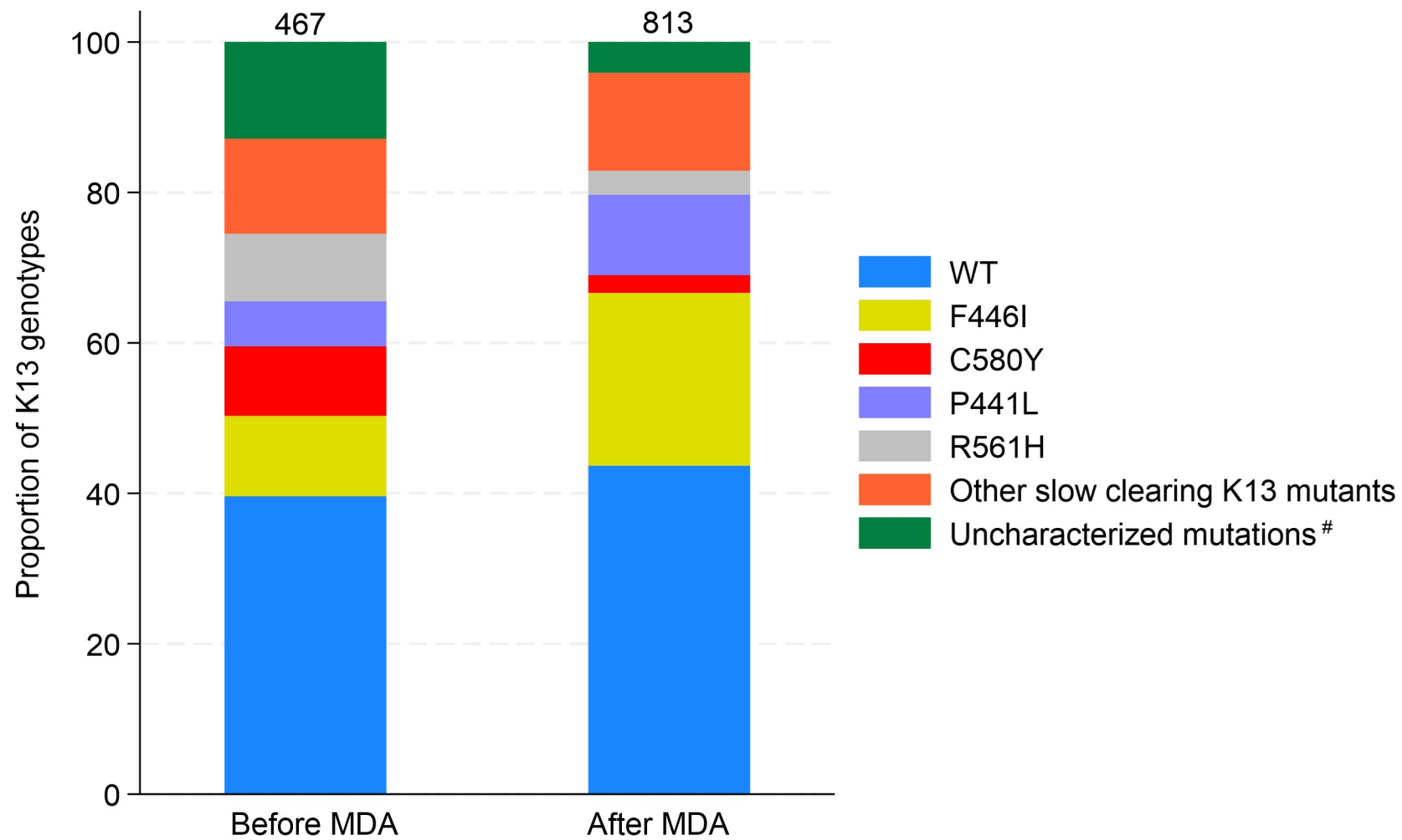

Supplement: Supplementary file 2 — Additional file 2: Figure S2. Comparison of the proportion K13 mutations before and after MDA. # Uncharacterized mutations are K13 mutations where the association with parasite clearance has not yet been established. [file 12936_2024_4955_MOESM2_ESM.pdf]
