# Supplementary material for: Molecular markers of artemisinin resistance during falciparum malaria elimination in Eastern Myanmar
Source: Malar J. 2024 May 8;23:138. doi: 10.1186/s12936-024-04955-6 (PMC11078751; doi:10.1186/s12936-024-04955-6)
Supplement: Supplementary file 3 — Additional file 3: Table S1A. K13 mutations frequency in Hpapun township from 2013 to 2019. Table S1B. K13 mutations frequency in Kyainseikgyi township from 2015 to 2017. Table S1C. K13 mutations frequency in Myawaddy, Kawkareik and Hlaingbwe townships from 2013 to 2019. Table S2. Status of uncharacterized K13 mutants in other contexts. [file 12936_2024_4955_MOESM3_ESM.docx]

**Additional Tables**

Supplementary Table 1A. K13 mutations frequency in Hpapun township from 2013 to 2019

|  |  | 2013 | | 2014 | | 2015 | | 2016 | | 2017 | | 2018 | | 2019 | | Total | |
| --- | --- | --- | --- | --- | --- | --- | --- | --- | --- | --- | --- | --- | --- | --- | --- | --- | --- |
|  |  | N | (%) | N | (%) | N | (%) | N | (%) | N | (%) | N | (%) | N | (%) | N | (%) |
| Wild type |  | 49 | (52.1) | 98 | (38.6) | 8 | (29.6) | 264 | (46.2) | 578 | (40.3) | 399 | (44.5) | 52 | (36.9) | 1,448 | (42.4) |
| Kelch13 mutations |  | 45 | (47.9) | 156 | (61.4) | 19 | (70.4) | 308 | (53.8) | 855 | (59.7) | 497 | (55.5) | 89 | (63.1) | 1,969 | (57.6) |
| E252Q^µ^ |  | 1 | (1.1) | 26 | (10.2) | - |  | 2 | (0.3) | 1 | (0.1) | 13 | (1.5) | - |  | 43 | (1.3) |
| P441L^µ^ |  | - |  | 6 | (2.4) | 8 | (29.6) | 65 | (11.4) | 263 | (18.4) | 91 | (10.2) | 41 | (29.1) | 474 | (13.9) |
| F446I^µ^ |  | 21 | (22.3) | 27 | (10.6) | - |  | 70 | (12.2) | 226 | (15.8) | 151 | (16.9) | 26 | (18.4) | 521 | (15.3) |
| G449A^µ^ |  | - |  | 9 | (3.5) | - |  | 42 | (7.3) | 122 | (8.5) | 84 | (9.4) | 6 | (4.3) | 263 | (7.7) |
| N458Y^µ^ |  | - |  | - |  | - |  | - |  | 1 | (0.1) | - |  | - |  | 1 | (0.0) |
| M476I^µ^ |  | 9 | (9.6) | 16 | (6.3) | - |  | 23 | (4.0) | 40 | (2.8) | 5 | (0.6) | - |  | 93 | (2.7) |
| N537I^µ^ |  | 1 | (1.1) | - |  | - |  | - |  | - |  | - |  | - |  | 1 | (0.0) |
| G538V^µ^ |  | - |  | 1 | (0.4) | - |  | 3 | (0.5) | 5 | (0.3) | - |  | - |  | 9 | (0.3) |
| R539T^µ^ |  | - |  | 1 | (0.4) | - |  | - |  | - |  | - |  | - |  | 1 | (0.0) |
| P553L^µ^ |  | - |  | - |  | - |  | 1 | (0.2) | - |  | - |  | - |  | 1 | (0.0) |
| R561H^µ^ |  | 8 | (8.5) | 24 | (9.4) | 2 | (7.4) | 39 | (6.8) | 126 | (8.8) | 111 | (12.4) | 13 | (9.2) | 323 | (9.5) |
| P574L^µ^ |  | - |  | 4 | (1.6) | - |  | - |  | 3 | (0.2) | 2 | (0.2) | - |  | 9 | (0.3) |
| C580Y^µ^ |  | 3 | (3.2) | 13 | (5.1) | - |  | 13 | (2.3) | 26 | (1.8) | 10 | (1.1) | 1 | (0.7) | 66 | (1.9) |
| P667T^µ^ |  | - |  | - |  | - |  | - |  | 2 | (0.1) | - |  | - |  | 2 | (0.1) |
| Uncharacterized mutations^#^ |  | 2 | (2.1) | 29 | (11.4) | 9 | (33.3) | 50 | (8.7) | 40 | (2.8) | 30 | (3.3) | 2 | (1.4) | 162 | (4.7) |

^µ^ slow parasites clearance K13 mutants

^#^ K13 mutations of which the association with parasite clearance has not yet been established

Supplementary Table 1B. K13 mutations frequency in Kyainseikgyi township from 2015 to 2017

|  | 2015 | | 2016 | | 2017 | | Total | |
| --- | --- | --- | --- | --- | --- | --- | --- | --- |
|  | N | (%) | N | (%) | N | (%) | N | (%) |
| Wild type | 156 | (29.2) | 69 | (14.9) | 143 | (33.4) | 368 | (25.8) |
| Kelch13 mutations | 379 | (70.8) | 393 | (85.1) | 285 | (66.6) | 1057 | (74.2) |
| E252Q^µ^ | 2 | (0.4) | - |  | - |  | 2 | (0.1) |
| P441L^µ^ | 2 | (0.4) | 1 | (0.2) | - |  | 3 | (0.2) |
| F446I^µ^ | 178 | (33.3) | 283 | (61.3) | 164 | (38.3) | 625 | (43.9) |
| G449A^µ^ | 28 | (5.2) | 34 | (7.4) | 16 | (3.7) | 78 | (5.5) |
| M476I^µ^ | 9 | (1.7) | 3 | (0.6) | 2 | (0.5) | 14 | (1.0) |
| N537I^µ^ | 19 | (3.6) | 11 | (2.4) | 13 | (3.0) | 43 | (3.0) |
| G538V^µ^ | 3 | (0.6) | - |  | - |  | 3 | (0.2) |
| R539T^µ^ | 1 | (0.2) | - |  | - |  | 1 | (0.1) |
| P553L^µ^ | 1 | (0.2) | 7 | (1.5) | 5 | (1.2) | 13 | (0.9) |
| R561H^µ^ | 4 | (0.7) | 3 | (0.6) | 5 | (1.2) | 12 | (0.8) |
| P574L^µ^ | 14 | (2.6) | 3 | (0.6) | 5 | (1.2) | 22 | (1.5) |
| C580Y^µ^ | 74 | (13.8) | 19 | (4.1) | 44 | (10.3) | 137 | (9.6) |
| Uncharacterized mutations^#^ | 44 | (8.2) | 29 | (6.3) | 31 | (7.2) | 104 | (7.3) |

^µ^ slow parasites clearance K13 mutants

^#^ K13 mutations of which the association with parasite clearance has not yet been established

Supplementary Table 1C. K13 mutations frequency in Myawaddy, Kawkareik and Hlaingbwe townships from 2013 to 2019

|  | 2013 | | 2014 | | 2015 | | 2016 | | 2017 | | 2018 | | 2019 | | Total | |
| --- | --- | --- | --- | --- | --- | --- | --- | --- | --- | --- | --- | --- | --- | --- | --- | --- |
|  | N | (%) | N | (%) | N | (%) | N | (%) | N | (%) | N | (%) | N | (%) | N | (%) |
| Wild type | 25 | (13.2) | 11 | (35.5) | 5 | (50.0) | 3 | (50.0) | 8 | (30.8) | 12 | (24.0) | 1 | (12.5) | 65 | (20.3) |
| Kelch13 mutations | 164 | (86.8) | 20 | (64.5) | 5 | (50.0) | 3 | (50.0) | 18 | (69.2) | 38 | (76.0) | 7 | (87.5) | 255 | (79.7) |
| E252Q^µ^ | 9 | (4.8) | 3 | (9.7) | 1 | (10.0) | - |  | - |  | - |  | - |  | 13 | (4.1) |
| P441L^µ^ | 4 | (2.1) | 3 | (9.7) | - |  | 1 | (16.7) | 8 | (30.8) | 3 | (6.0) | - |  | 19 | (5.9) |
| F446I^µ^ | - |  | - |  | - |  | - |  | 1 | (3.8) | 8 | (16.0) | 1 | (12.5) | 10 | (3.1) |
| G449A^µ^ | - |  | - |  | - |  | - |  | 1 | (3.8) | - |  | - |  | 1 | (0.3) |
| N458Y^µ^ | 6 | (3.2) | - |  | - |  | - |  | - |  | - |  | - |  | 6 | (1.9) |
| M476I^µ^ | 8 | (4.2) | - |  | - |  | - |  | - |  | - |  | - |  | 8 | (2.5) |
| A481V^µ^ | 1 | (0.5) | - |  | - |  | - |  | - |  | - |  | - |  | 1 | (0.3) |
| N537I^µ^ | 2 | (1.1) | - |  | - |  | - |  | - |  | - |  | - |  | 2 | (0.6) |
| G538V^µ^ | 20 | (10.6) | 3 | (9.7) | - |  | - |  | - |  | - |  | - |  | 23 | (7.2) |
| R561H^µ^ | 7 | (3.7) | - |  | - |  | - |  | 2 | (7.7) | 3 | (6.0) | - |  | 12 | (3.8) |
| P574L^µ^ | 9 | (4.8) | 4 | (12.9) | 1 | (10.0) | - |  | - |  | - |  | - |  | 14 | (4.4) |
| C580Y^µ^ | 83 | (43.9) | 5 | (16.1) | 1 | (10.0) | 2 | (33.3) | 1 | (3.8) | 12 | (24.0) | - |  | 104 | (32.5) |
| A675V^µ^ | 3 | (1.6) | 2 | (6.5) | 1 | (10.0) | - |  | - |  | - |  | - |  | 6 | (1.9) |
| Uncharacterized mutations^#^ | 12 | (6.3) | - |  | 1 | (10.0) | - |  | 5 | (19.2) | 12 | (24.0) | 6 | (75.0) | 36 | (11.3) |

^µ^ slow parasites clearance K13 mutants

^#^ K13 mutations of which the association with parasite clearance has not yet been established see more details in supplementary table 2.

Supplementary table 2 Status of uncharacterized K13 mutants in other contexts

| K13 mutations | N | % | Status in other contexts | References |
| --- | --- | --- | --- | --- |
| C469F/Y | 73 | 24.2 | Mutant reported previously* but unknown effect on clearance | ^1, 2, 3, 4^ |
| M562I | 64 | 21.2 | unknown effect on clearance |  |
| G533S | 54 | 17.9 | unknown effect on clearance |  |
| K189T | 47 | 15.6 | Not associated with delayed clearance | ^5^ |
| K438N | 11 | 3.6 | unknown effect on clearance |  |
| R575K | 10 | 3.3 | Associated with delayed clearance | ^2^ |
| K479I | 6 | 2.0 | Associated with delayed clearance | ^2^ |
| D584V | 5 | 1.7 | Associated with delayed clearance | ^2^ |
| I205T | 3 | 1.0 | No information about this mutant reported elsewhere |  |
| E321K | 2 | 1.0 | No information about this mutant reported elsewhere |  |
| F614L | 2 | 0.7 | No information about this mutant reported elsewhere |  |
| G533A | 2 | 0.7 | No information about this mutant reported elsewhere |  |
| V534G | 2 | 0.7 | No information about this mutant reported elsewhere |  |
| A621V | 1 | 0.7 | No information about this mutant reported elsewhere |  |
| A626S | 1 | 0.3 | No information about this mutant reported elsewhere |  |
| C542Y | 1 | 0.3 | No information about this mutant reported elsewhere |  |
| D109Y | 1 | 0.3 | No information about this mutant reported elsewhere |  |
| D452E | 1 | 0.3 | Associated with delayed clearance | ^2^ |
| E208K | 1 | 0.3 | Not associated with delayed clearance | ^5^ |
| G533D | 1 | 0.3 | No information about this mutant reported elsewhere |  |
| G718S | 1 | 0.3 | No information about this mutant reported elsewhere |  |
| K586E | 1 | 0.3 | Mutant reported previously, but unknown effect on clearance | ^6^ |
| N264I | 1 | 0.3 | No information about this mutant reported elsewhere |  |
| N490H | 1 | 0.3 | No information about this mutant reported elsewhere |  |
| N525Y | 1 | 0.3 | No information about this mutant reported elsewhere |  |
| R265P | 1 | 0.3 | No information about this mutant reported elsewhere |  |
| R528S | 1 | 0.3 | No information about this mutant reported elsewhere |  |
| R529G | 1 | 0.3 | No information about this mutant reported elsewhere |  |
| S423N | 1 | 0.3 | No information about this mutant reported elsewhere |  |
| T192I | 1 | 0.3 | No information about this mutant reported elsewhere |  |
| T535M | 1 | 0.3 | No information about this mutant reported elsewhere |  |
| V193E | 1 | 0.3 | No information about this mutant reported elsewhere |  |
| V494I | 1 | 0.3 | No information about this mutant reported elsewhere |  |

*Increased survival in in-vitro assay

1. Siddiqui, F. A., Boonhok, R., Cabrera, M., Mbenda, H. G. N., Wang, M., Min, H., ... & Cui, L. (2020). Role of Plasmodium falciparum Kelch 13 protein mutations in P. falciparum populations from northeastern Myanmar in mediating artemisinin resistance. MBio, 11(1), 10-1128.
2. Owoloye, A., Olufemi, M., Idowu, E. T., & Oyebola, K. M. (2021). Prevalence of potential mediators of artemisinin resistance in African isolates of Plasmodium falciparum. Malaria journal, 20, 1-12
3. Tumwebaze, P. K., Conrad, M. D., Okitwi, M., Orena, S., Byaruhanga, O., Katairo, T., ... & Rosenthal, P. J. (2022). Decreased susceptibility of Plasmodium falciparum to both dihydroartemisinin and lumefantrine in northern Uganda. Nature communications, 13(1), 6353.
4. Ye, S. Y., Ye, R., Li, C. F., Tang, Y. R., & Zhou, H. N. (2021). Polymorphisms of the K13 gene of Plasmodium falciparum in the China-Myanmar border area.
5. Ndwiga, L., Kimenyi, K. M., Wamae, K., Osoti, V., Akinyi, M., Omedo, I., ... & Ochola-Oyier, L. I. (2021). A review of the frequencies of Plasmodium falciparum Kelch 13 artemisinin resistance mutations in Africa. International Journal for Parasitology: Drugs and Drug Resistance, 16, 155-161.
6. Lautu-Gumal, D., Razook, Z., Koleala, T., Nate, E., McEwen, S., Timbi, D., ... & Barry, A. E. (2021). Surveillance of molecular markers of Plasmodium falciparum artemisinin resistance (kelch13 mutations) in Papua New Guinea between 2016 and 2018. International Journal for Parasitology: Drugs and Drug Resistance, 16, 188-193.

Supplementary text

Text 1 Laboratory methods

1. Sanger sequencing. Polymorphisms in the K13 gene were assessed by nested PCR amplification covering the propeller region of the gene, followed by sequencing using an ABI Sequencer (Macrogen Inc, South Korea).
2. Illumina sequencing**.** DNA samples with more than 200 copies of *P. falciparum* genome per ul were processed for selective whole genome amplification (sWGA) and deep sequencing, as described in (Oyola et al., 2016).
